# From the Patient's Point of View

**Published:** 1921-05-28

**Authors:** 


### From the Patient's Point of View.

### A SUGGESTION TO THE COMMISSIONER OF POLICE.

Someone has endeavoured to average the length of time a person spends in sleep during his lifetime as one-third, and, having been a patient for some time past in a well-known London hospital in the West Central district, I suppose my acquaintance with somnolence should be above par. But there were times when I felt that, through no fault of mine or the hospital authorities, I was cheated out of my full share of rest and quiet.

This particular institution happens to be situated in the busiest part of a busy city, where each day, from morning until evening, year in and year out, arises the unceasing roar of traffic. The following carefully compiled tables may be of interest as showing the average number of vehicles passing in the street at one side only of the hospital per hour per diem:—

| | |
|---|---|
| Taxis | 312 |
| Tricycle carriers | 11 |
| Motor cars, private | 13 |
| Number of motor vehicles | 336 |
| Carts, vans, etc. (carrying merchandise, etc.) | 96 |
| Coal carts * | 22 |
| Station broughams, etc. | 14 |
| Number of horse-drawn vehicles | 132 |

From experience I can also write that the noise during the night is heaviest between the hours of 1 and 2 a.m.; the lightest interval in one week being the early hours of Sunday morning. Because of the proximity of Covent Garden market this street is specially used by incoming traffic from suburban and outlying country districts. One special contrivance belonging, I believe, to the City of London, or to the responsible authorities for street cleaning, was especially annoying. Should one happen to be merely wakeful and not in any pain its resemblance to a vigorously conducted Jazz band may have been soothingly reminiscent of the outside world and all that therein is; but if the sufferer was in actual pain when the conveyance passed he promptly decided there could be nothing like it on earth. At such a time the patient could almost wish the drummer would develop encephalitis lethargica (severe) immediately before his next evening performance.

And here is food for thought for our ever-ready Commissioner of Police—the desirability of diverting the greater part of the traffic into near-by streets where it is particularly heavy around our hospitals in the London area. The problem should be more interesting than difficult to him. It is his duty to make special traffic regulations to assist the opening of Parliament, Coronations, and defeat the aims of Sinn Fein. Even for public funerals of policemen traffic has been diverted. Why not assist the living?

L.

* With their accompanying stentorian criers.

